# Black carbon radiative forcing at TOA decreased during aging

**DOI:** 10.1038/srep38592

**Published:** 2016-12-05

**Authors:** Yu Wu, Tianhai Cheng, Lijuan Zheng, Hao Chen

**Affiliations:** 1 State Key Laboratory of Remote Sensing Science, Institute of Remote Sensing and Digital Earth, Chinese Academy of Sciences, China

## Abstract

During aging processing, black carbon (also called soot) particles may tend to be mixed with other aerosols, and highly influence their radiative forcing. In this study, freshly emitted soot particles were simulated as fractal aggregates composed of small spherical primary monomers. After aging in the atmosphere, soot monomers were coated by a thinly layer of sulfate as thinly coated soot particles. These soot particles were entirely embedded into large sulfate particle by further aging, and becoming heavily coated soot particles. In clear-sky conditions, black carbon radiative forcing with different aging states were investigated for the bottom and top of atmosphere (BOA and TOA). The simulations showed that black carbon radiative forcing increased at BOA and decreased at TOA after their aging processes. Thinly and heavily coated states increased up to ~12% and ~35% black carbon radiative forcing at BOA, and black carbon radiative forcing at TOA can reach to ~20% and ~100% smaller for thinly and heavily coated states than those of freshly emitted states, respectively. The effect of aging states of black carbon radiative forcing was varied with surface albedo, aerosol optical depth and solar zenith angles. These findings would be helpful for the assessments of climate change.

Solar absorption and scattering by black carbon is one of the critical issues with large uncertainties in climate change studies^1^. As main sources of anthropogenic atmospheric aerosols, black carbon (BC, also called soot or light absorbing carbon) particles are mainly emitted from the incomplete combustion of fossil fuels and biomass burning, and become one of the most important components of global warming in terms of direct forcing^2^. Mass absorption strength and scattering ability of an aerosol particle strongly depends on the particle effective size and mixing state^3^,^4^. Both of those quantities depend on the size and chemical composition of black carbon as it is released into the atmosphere as well as its micro- physical and chemical evolution during transport through the atmosphere^5^,^6^. During atmospheric processing, soot particles tend to be internally mixed with other weak or non-absorbing materials, such as sulfate, organics, dust, and sea salt^7^,^8^,^9^. The radiative forcing contribution of these light absorbing aerosols is still quite uncertain in climate forcing assessments because of the incomplete understanding of the effect of aging states on the radiative properties of soot aerosols^10^,^11^.

The direct radiative forcing of aerosols on the atmospheric radiation balance of the Earth mainly depends on their optical properties^12^,^13^. The variation of single scattering albedo can modify the sign of the aerosol radiative forcing (cooling/heating, depending on the planetary albedo), while the asymmetry parameter of the phase function together with aerosol loading can drive the magnitude of aerosol radiative forcing^14^,^15^. Previous studies indicated that the extinction coefficient, single scattering albedo and asymmetry parameter of soot aerosols may be significantly affected by their mixing states^16^,^17^. Previous laboratory experiments showed that the embedding of soot aggregates in weakly absorbing particles leads to the enhancement of light absorption and scattering^18^. This amplification of the absorption cross-section for soot internally mixed (or coated) with sulfate or organics was measured up to ~150−200%^19^. The radiative forcing at the top of the atmosphere (TOA) was simulated as two times higher if fresh black carbon is modeled as an aggregate instead of a homogeneous sphere^20^. The radiative forcing is also ~20% less when modeling internally mixed BC particles as embedded lacy aggregates than with a simple core-shell shape, which is the shape assumed in many climate models^21^. These studies indicate that it is necessary to account for the radiative effects of morphological differences and aging states of aerosols in climate modeling^22^.

Based on the transmission electron microscopy (TEM) and scanning electron microscopy (SEM) images, the freshly emitted soot aerosols were aggregated by hundreds of tiny monomers (primary particles)^23^,^24^. The radiative properties of soot aggregates are mainly influenced by complex morphological and chemical parameters, such as fractal structure, monomer character, mixing state, and refractive index^25^,^26^,^27^. Results of *in situ* measurements and laboratory studies indicate that freshly emitted BC particles tend to be coated with a thin layer of other aerosol components in the atmosphere through the coagulation and condensation of secondary aerosol compounds^28^. With the aging of the light absorbing carbon particles, most BC particles are thickly coated and tend to be compact^29^,^30^. Morphology of internally mixed soot aerosols is complex, depending highly on the degree of aging, ambient temperature, and relative humidity^31^. Optical properties of aged soot particles were obviously changed for these different morphological and chemical variations^32^,^33^,^34^,^35^.

The radiative properties of soot aerosols in climate models are commonly obtained based on the morphological simplification of homogenous spheres for freshly emitted states and the single core-shell spheres for aged states using Lorenz-Mie-Debye theory^36^,^37^. However, large discrepancies have been measured and simulated between the aggregates and the equivalent sphere approximations due to their complex morphologies, components and multiple scattering^38^,^39^,^40^.

To quantify the effect of aging states on the radiative forcing of soot aerosols, the numerically exact superposition T-matrix (STM) method was used to reconstruct the absorption and scattering properties of these heterogeneous mixtures composed of fractal aggregated soot particles and non-absorbing aerosol particles with different aging states. The advantage of the STM method is that it is a direct computer solver of the frequency-domain macroscopic Maxwell equations and is numerically exact^41^,^42^. This method is highly efficient and accurate and can be used to study the finest details of electromagnetic scattering patterns, which are unattainable with other techniques^43^. The libradtran software package was further used for the radiative transfer calculations of the black carbon for different aging states. Libradtran allows to compute (polarized) radiances, irradiances, and actinic fluxes in the solar and thermal spectral regions^44^. It is an effective tool for climate studies, e.g., for the calculation of radiative forcing due to different atmospheric components, and remote sensing of clouds, aerosols and trace gases in the Earth’s atmosphere^45^.

## Results and Discussion

### Black carbon optical properties for different aging states

As soot particles formed and transported in the atmosphere, the individual freshly emitted soot particles tend to be coated with other non-absorbing materials. The aging process of soot aerosols results in a dramatic change in the morphological parameters^46^,^47^,^48^. Freshly emitted soot particles consist of small spherical primary particles combined into branched aggregates, the soot monomers are coated by a thin layer of other aerosol components as thinly coated soot particles, and the soot particles are entirely embedded into other aerosol components in heavily coated soot particles. Compared with freshly emitted soot particles, each monomer of thinly coated soot particles can be assumed as having structures of a concentric core containing black carbon particles with high light absorption and a shell containing weakly absorbing particles^49^. In such cases, morphology of soot particle alters to compact closed structures, and the morphology of other materials coated on the soot particles is simply assumed as homogeneous spheres. The other material coating the soot particles is assumed to be sulfate in this study.

Calculations of the optical properties of soot aerosols are usually performed by computing single-particle optical properties based on the physical and chemical properties of the particles, followed by performing an ensemble average over morphologies, sizes, and compositions. To quantify and study the impact of aging states on the multi-scattering properties of soot aerosols, models of the radiative properties of soot aerosols were simply assumed to be mixtures composed of two components (soot and sulfate) with fixed volumes. In this study, the soot volume fractions (*F*_*soot*_) of these aerosols were used to indicate the volume ratios of soot to the entire soot-sulfate mixtures. For freshly emitted soot aerosols, the soot volume fractions were assumed to be 1, and decreased to 2/3 and 1/3 for thinly and heavily coated states of aerosol ensembles, in this study.

**Figure 1:**
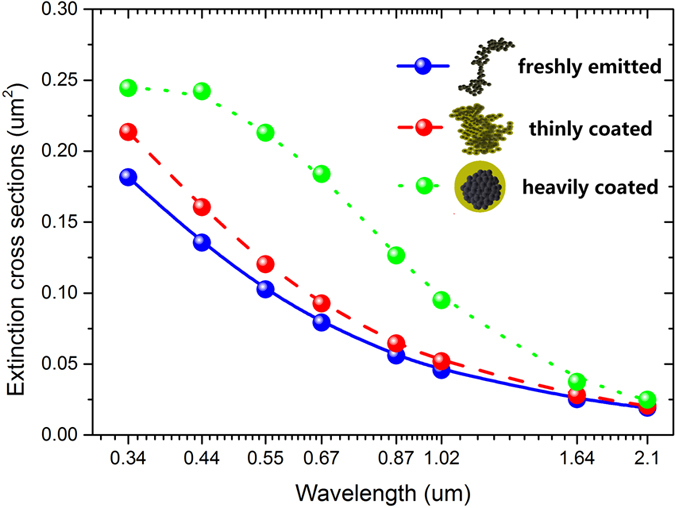
Extinction cross sections (Cext) of soot aerosols with different aging states (freshly emitted, thinly coated and heavily coated).

As shown in Fig. 1, during the aging processes of soot aerosols, their optical properties were dramatically changed due to the effects of morphology such as the formation of coated shell and compaction of BC particles in the shortwave spectral. The cross sections of extinction, absorption and scattering of aged soot aerosols were monotonously larger than the freshly emitted soot aerosols with their aging states. For example of 0.67 μm, it is shown from Fig. 1 that the extinction cross sections of heavily coated soot aerosols were twice of the freshly emitted soot aerosols, while the thinly coated soot aerosols were ~20% larger than the fresh states. Absorption cross sections of thinly coated soot aerosols were slightly (~10%) larger than freshly emitted soot aerosols, and it significantly increased to ~60% for the heavily coated states. This absorption enhancement of the ratio of ~160% is agreed with previous simulations and observations. It is reported that the ratio of absorption between soot coated with large sulfate and fresh soot was ~150–200% in the visible range^19^,^40^. Scattering enhancements of aging processes were significantly larger than their absorption enhancements. Thus, different mixing states of soot aerosols resulted in significant changes in the single scattering albedo. At 0.67 μm, ratio of SSA between the thinly coated and the freshly emitted soot aerosols were ~150%, and it grown to ~250% in the heavily coated states. With the aging processes, the soot volume fractions were largely decreased due to the attachments of sulfate particles, thus, the single scattering albedos of the soot aerosols were increased.

### Black carbon radiative forcing for different aging states

With the aging of soot aerosols, freshly emitted soot aerosol are tend to be compact and thickly coated. To investigate and quantify the effects of the aging state on the radiative forcing of aerosols mixtures, different solar zenith angles (SZA, 30, 40, 50, 60, and 70 degree), surface albedos (0, 0.1, 0.2, 0.4, 0.6, and 0.9), and aerosol optical depth at 0.55 μm (AOD_0.55_, 0.1, 0.2, 0.3, 0.5, 0.7, and 1.0) were considered. The radiative forcing of soot aerosols on both BOA and TOA were calculated using DISORT radiative transfer model. In these different scenarios, the radiative properties of soot aerosols were analyzed.

**Figure 2:**
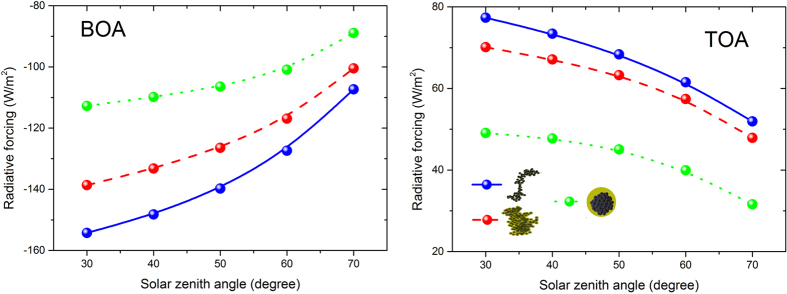
Black carbon radiative forcing for different aging states (freshly emitted, thinly coated and heavily coated) at BOA and TOA for different solar zenith angles (30, 40, 50, 60, and 70 degrees). AOD_0.55_ = 0.3, Albedo = 0.2.

It can be seen from Fig. 2 that larger solar zenith angles (*θ*) may introduce larger radiative forcing at BOA and smaller radiative forcing at TOA. The solar radiation is proportional to the solar constant and the cosine of the solar zenith angle 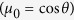, thus, the downward irradiances were smaller for larger solar zenith angles, which leads to the decline of magnitude of radiative forcing. For radiative forcing at BOA, it is the difference between the net downward irradiances of cloud-free atmospheres with and without aerosols. The radiative forcing at TOA is the difference between upward irradiances of cloud-free atmospheres without and with aerosols. The differences of radiative forcing between different aging states were decreased by larger solar zenith angles mainly due to the decline of solar radiations. Radiative forcing of heavily coated black carbon at BOA were varied from −112 W/m^2^ (negative, 30 degree) to −89 W/m^2^ (negative, 70 degree), while those at TOA were 49 W/m^2^ and 32 W/m^2^, respectively. The solar zenith angle changes on both diurnal and seasonal time scales, greatly influencing the total solar radiation that is available to interact with black carbon aerosols.

After aging processes, black carbon radiative forcing increases at BOA and deceases at TOA. In general, black carbon radiative forcing were negative for BOA, and were often positive for TOA. Ramana *et al*.^6^ implied that the atmospheric ratio of black carbon to sulfate exerted a strong positive influence on the net warming, and showed that solar-absorption efficiency was positively correlated with the ratio of black carbon to sulfate^6^. The simulation indicated similar results that smaller ratio of black carbon to sulfate (more aged states) leads to smaller radiative forcing at TOA, because the black carbon aerosols with larger single scattering albedo enhance the multiple scattering of surface and aerosol and thus perform larger upward irradiance at TOA. The aging processes of black carbon aerosols decreases their radiative forcing at TOA in a monotonous way. For example of solar zenith angle equals 50 degree, the black carbon radiative forcing at TOA was ~70 W/m^2^. After aging, it decreased to ~64 W/m^2^ for thinly coated states, and it further reduced to ~48 W/m^2^ for heavily coated states. It is implied that the black carbon aging states should be considered to be an important influence factor on their radiative forcing estimations.

The effect of aging states on the radiative forcing of fractal aggregated black carbon aerosols was weakened by the augment of solar zenith angles. In this study, we defined the relative deviations of black carbon aerosol radiative forcing after their aging processes 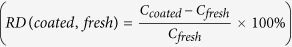 in percentage to indicate the effects of aging states on the radiative forcing of fractal aggregated black carbon aerosols. At BOA, relative deviations between thinly coated and freshly emitted soot aerosols were ~−10% (negative), and these relative deviations varied to be ~−25% (negative) for heavily coated soot aerosols in the cases of solar zenith angle equals 30 degree. At TOA, these relative deviations between thinly coated and freshly emitted soot aerosols were ~−8% (negative), and it reached to ~−40% (negative) for heavily coated soot aerosols. The results showed that these relative deviations were slightly decreased (~5–10%) with larger solar zenith angles. The aging of black carbon aerosols increases their single scattering albedos, and it accordingly amplified the multiple scattering of surface and aerosol. Stronger multiples scattering intensifies the net downward irradiances at BOA and upward irradiances at TOA. Black carbon aging reduces the differences of net downward irradiances at BOA and upward irradiances at TOA between the cloud-free atmospheres without aerosols and with freshly emitted black carbon aerosols. As results, the augment of solar zenith angle decreases the relative deviations of radiative forcing between different aging states.

**Figure 3:**
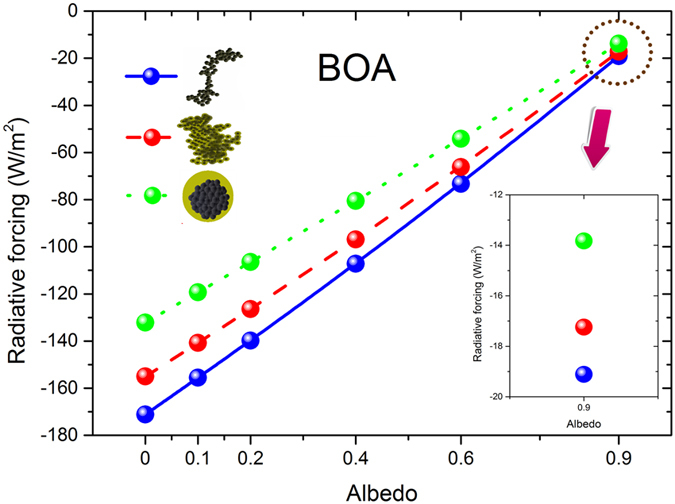
Black carbon radiative forcing for different aging states (freshly emitted, thinly coated and heavily coated) at BOA for different surface albedo (0, 0.1, 0.2, 0.4, 0.6, and 0.9). AOD_0.55_ = 0.3, SZA = 50°.

**Figure 4:**
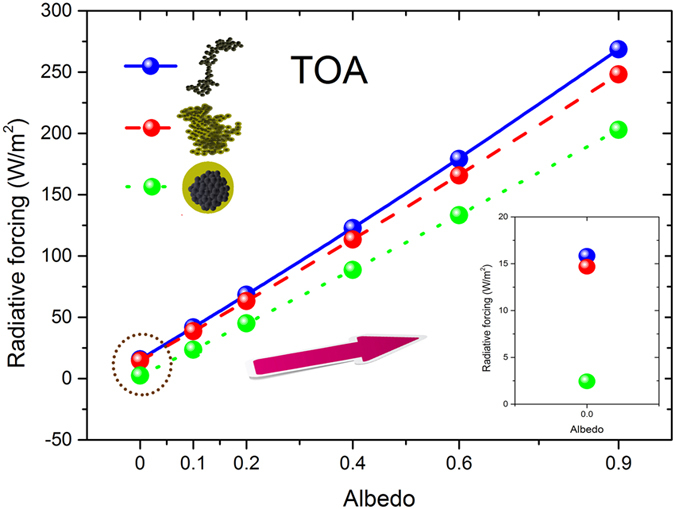
Similar as Fig. 3, but at TOA.

Figures 3 and 4 showed the black carbon radiative forcing with different aging states and variable Lambertian surface albedo (*α*_*s*_) at BOA and TOA, respectively. Black carbon radiative forcing at BOA and TOA were increased with higher surface albedo. At BOA, the downward irradiances of the conditions with aerosols are smaller than those conditions without aerosols. The augment of surface albedo leads to more upward irradiances at surface and tend to reduce the differences of net downward irradiances between the conditions with and without aerosols. Therefore, when surface albedo increases, the magnitude of black carbon radiative forcing at BOA decreases (smaller negative value). Black carbon radiative forcing at TOA also increases with higher surface albedo because more radiations reflected from surface and absorbed again by black carbon aerosols before it reaches to TOA.

The differences of black carbon radiative forcing between different aging states decrease at BOA and increase at TOA with higher surface albedo. At BOA, due to multiple scattering of surface and aerosol, higher surface albedo slightly increased the diffuse downward irradiances and largely increased the diffuse upward irradiances. The aging of black carbon aerosols amplified the multiple scattering of surface and aerosol due to the augment of their single scattering albedo. Therefore, the differences of net downward irradiances between different aging states were narrowed with higher surface albedo at BOA. Moreover, higher surface albedo leads to larger radiation reflected from surface to TOA, and thus the differences of black carbon radiative forcing between different aging states were amplified at TOA.

Black carbon radiative forcing increases at BOA and decreases at TOA by their aging for various surface albedos. The aging of black carbon aerosols narrowed the differences of radiative forcing between the conditions with and without aerosols, because the augment of single scattering albedo intensified the net downward irradiances at BOA and the upward irradiances at TOA. The results illustrate that the relative deviations of black carbon radiative forcing between different aging states were ~−10% (negative) for thinly coated states, and it varied to ~−28% (negative) for heavily coated states at BOA, in the cases of AOD_0.55_ = 0.3, SZA = 50°. Among the simulations, the largest relative difference can reach ~−12% (negative) and ~−35% (negative) for thinly and heavily coated states, respectively. At TOA, the relative deviations between thinly coated and freshly emitted soot aerosols were ~−8% (negative), and further aging leads to the amplifications of the relative deviations between heavily coated and freshly emitted soot aerosols (more than ~−30%, negative). These largest relative deviations at TOA can reach to ~−20% (negative) and ~−100% (negative) for thinly and heavily coated states, respectively.

The effect of black carbon aging on their radiative forcing was remarkably influenced by the surface albedo. At TOA, the radiative forcing of heavily coated soot aerosols was significantly (~85%) smaller than those of freshly emitted soot aerosols when surface albedo equals 0.0. For higher surface albedo, these relative deviations between heavily coated and freshly emitted soot aerosols were considerably smaller, for instance, ~25% in the case of surface albedo equals 0.9. Among the simulations, the relative deviations between heavily coated and freshly emitted black carbon aerosols were varied from to 80~100% (*α*_*s*_ = 0) to 15~30% (*α*_*s*_ = 0.9) at TOA, but the corresponding relative deviations were slightly (from −22% to −27%) enhanced for larger surface albedos at BOA. Higher surface albedo leads to stronger multiple scattering of surface and aerosol, and it slightly increased the diffuse downward irradiances and largely increased the diffuse upward irradiances. Due to larger single scattering albedo, the aged black carbon aerosols tend to generate more diffuse downward and upward irradiances than their freshly emitted states. The differences of diffuse upward irradiances from BOA to TOA between cloud-free atmospheres without aerosols and with freshly emitted black carbon aerosols were reduced by their aging. Therefore, the relative deviations of black carbon radiative forcing between different aging states remarkably reduces at TOA with higher surface albedo. At BOA, with higher surface albedo, the augment of diffuse upward irradiances was much more than the diffuse downward irradiances, which leads to more decline of the net downward irradiances by the black carbon aging. Therefore, the relative deviations of black carbon radiative forcing between different aging states were increased with higher surface albedos at BOA. It is implied that the effect of black carbon aging on their radiative forcing was sensitive to the surface albedo, thus, it was necessary to take into account this effect in climate studies.

**Figure 5:**
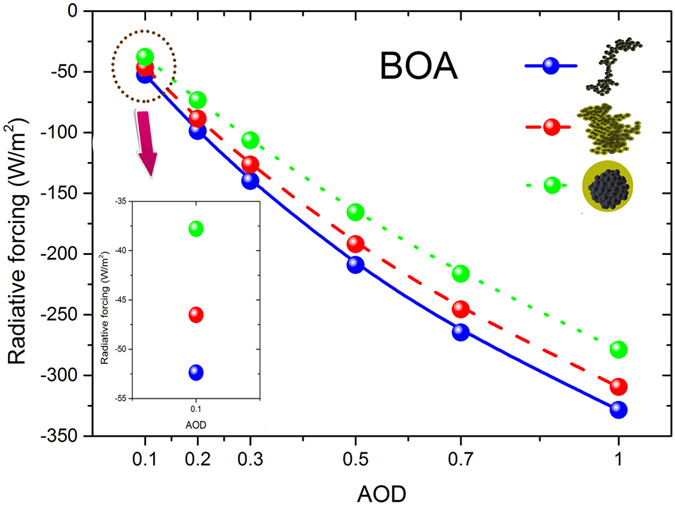
Black carbon radiative forcing for different aging states (freshly emitted, thinly coated and heavily coated) at BOA for different aerosol optical depth at 0.55 μm (0.1, 0.2, 0.3, 0.5, 0.7, and 1.0). Albedo = 0.2, SZA = 50°.

**Figure 6:**
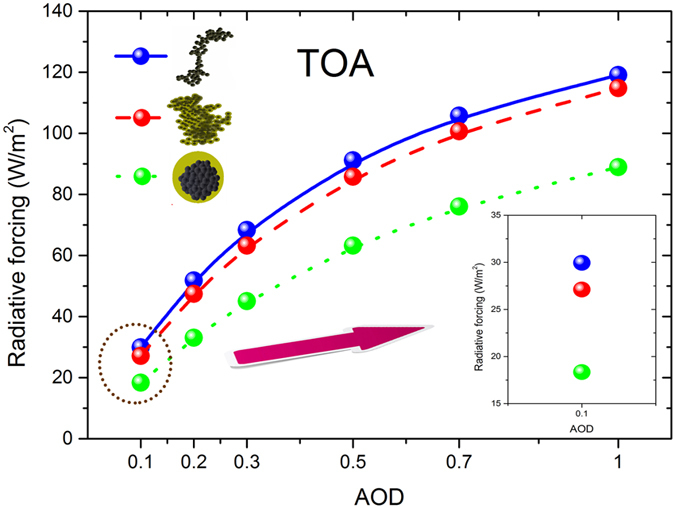
Similar as Fig. 5, but at TOA.

As shown in Figs 5 and 6, the black carbon radiative forcing with different aging states decreases at BOA and increases at TOA with larger black carbon aerosol loadings (aerosol optical depth). When the black carbon aerosol loading increases, the radiative differences of the atmosphere with and without black carbon aerosols enhances by an increase in path lengths or particle concentration or both, and the differences of radiative forcing between different aging states were also amplified due to their varied single scattering albedos.

Black carbon aging also leads to larger radiative forcing at BOA and smaller radiative forcing at TOA for different aerosol optical depths. It was assumed that the albedo is 0.2 and the solar zenith angle is 50 degree. The results showed that the relative deviations of black carbon radiative forcing between thinly coated and freshly emitted states were ~−10% (negative) at BOA, and the relative deviations between heavily coated and freshly emitted soot aerosols were amplified to ~−28% (negative) in the case of AOD_0.55_ = 0.1. At TOA, the relative deviations between thinly coated and freshly emitted soot aerosols can reach to ~−10% (negative), and the relative deviations between heavily coated and freshly emitted soot aerosols were magnified (up to ~−39% for the cases of AOD_0.55_ = 0.1, negative).

The effect of aging states on the radiative forcing of black carbon aerosols was weakened by larger black carbon aerosol loading. Relative deviations between aged and fresh black carbon aerosols were reduced for higher aerosol optical depths at both BOA and TOA. For example of AOD_0.55_ = 1.0, the black carbon radiative forcing with heavily coated states at BOA were ~15% smaller than those of freshly emitted soot aerosols, while these relative deviations were ~28% in the cases of AOD_0.55_ = 0.1. The variations of relative deviations between black carbon radiative forcing with different aging states caused by the aerosol optical depth were ~5–10% for thinly coated states, and ~10–20% for heavily coated sates. The net downward irradiances at BOA and upward irradiances at TOA were intensified by black carbon aging because of the amplified multiple scattering of surface and aerosol. Higher aerosol optical depth weakens the net downward irradiances at BOA and upward irradiances at TOA, thus it reduces the relative deviations of radiative forcing between different aging states.

The single scattering albedo of black carbon aerosols can be considered to be an important influence factor of their radiative forcing. During aging processes, the single scattering albedos of black carbon aerosols were largely increased by coating more organics materials and sulfate particles. Due to the larger single scattering albedo, the multiple scattering of surface and aerosol was amplified by the black carbon aging. With constant surface albedos and aerosol loadings, stronger multiples scattering may intensifies the net downward irradiances at surface and upward irradiances at TOA. It is implied that black carbon aging tends to reduce the differences of net downward irradiances at BOA and upward irradiances at TOA between the cloud-free atmospheres without aerosols and with freshly emitted black carbon aerosols. Therefore, black carbon radiative forcing commonly increases at BOA and decreases at TOA by their aging.

Previous studies also indicated that the radiative effect of aerosols was affected by the surface albedo, aerosol optical depth and single scattering albedo^14^,^50^. It is indicated that heavier dust aerosols may more heat the atmosphere and their radiative forcing at TOA were increased by larger surface albedo^51^. It is also shown in the previous studies of cloud particles that the radiative forcing becomes more sensitive to cloud microphysical composition, as the surface albedo decreases, the sun becomes higher in the sky, and the clouds attenuates^52^. The effect of aging states on black carbon radiative forcing was similarly influenced by these parameters. Relative deviations of radiative forcing at TOA between different aging states of black carbon aerosols were decreased with higher surface albedo (~50–80%), larger solar zenith angles (~5–10%), and higher aerosol optical depth (~10–20%) in these simulations. It is suggested that these important factors should be taken into account in the climate effect of black carbon aerosols.

Black carbon radiative forcing with different aging states were also compared to their single spherical approximations, which is commonly used in the climate studies. Freshly emitted soot particles were often simplified to be a volume-equivalent homogenous sphere, and the aged soot particles were assumed to be a single core-shell sphere model with equivalent soot and sulfate volumes. The relative deviations of black carbon radiative forcing between more realistic models of fractal aggregated soot particles and their morphological simplifications in percentage 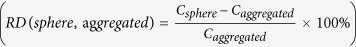 were calculated for both BOA and TOA. We found that the averaged radiative forcing of heavily coated soot aerosols were slightly overestimated by the single core-shell sphere model (~8% at BOA and ~5% at TOA, positive). However, the averaged radiative forcing of thinly coated soot aerosols were underestimated by the single core-shell sphere model (~−4% at BOA and ~−30% at TOA, negative). The morphological simplification of commonly used homogenous sphere may introduce large errors for the radiative forcing estimations of freshly emitted soot aerosols (~−12% at BOA and ~−40% at TOA, negative, on average). The absolute values of these relative deviations between the single homogenous sphere approximation and freshly emitted soot aerosols can reach to ~−100% or even larger in some conditions. Adachi *et al*.^21^ suggested that the radiative forcing is ~20% less when modeling internally mixed black carbon particles as embedded lacy aggregates than with a single core-shell sphere^21^. According to the observations, black carbon particles are thickly coated and tend to be compact with the aging of soot particles^29^,^48^. Due to the compact morphologies of soot aggregates, the relative deviations of radiative forcing between those aged soot aggregates and their single core-shell sphere approximations were limited. However, this morphological simplification may introduce large errors for freshly emitted soot aggregates with lacy morphologies. Among the simulations, the spherical approximations considerably underestimated the radiative forcing of freshly emitted and thinly coated soot aerosols. However, the radiative forcing of heavily coated soot aerosols were overestimated in a limited range. These findings should improve our understanding of the effects of aging states on the radiative properties of soot aerosols and their effects on climate.

## Methods

### Black carbon models for different aging states

The construction and morphology of these soot aggregates could be described by the well-known fractal law:


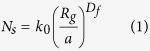



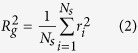


where *N_s_* is the number of monomers in the cluster, *a* is the mean radius of the monomers, *k*_0_ is the fractal prefactor, *D_f_* is the fractal dimension, *R_g_* is the radius of gyration, which represents the deviation of the overall aggregate radius in a cluster, and *r*_*i*_ is the distance from the ith monomer to the center of the cluster. The aging process of black carbon aerosols results in a dramatic change in morphological parameters. This morphological change can be described by an increase in the fractal dimension (*D_f_*  ). Previous measurements and simulations suggested the fractal dimensions of bare and coated BC particles varied from 1.8 to 3.0 with the fractal prefactor of 1.2^8^. Bond and Bergstrom^53^ reported the value of mean radius of BC monomer *a* in the range of 0.01–0.025 μm. In this study, the monomer number *N_s_* = 200, mean monomer radius *a* = 0.02 *μm*, and fractal prefactor *k*_0_ = 1.2 are assumed to be constant for the soot aggregates. The fractal dimension of soot aggregates were assumed to be 1.8, 2.4, and 3.0 for freshly emitted, thinly coated and heavily coated soot aerosols, respectively.

In the fresh cases, the soot monomers were semi-externally mixed with each other. This morphology was simplified and assumed to be a single homogeneous sphere with equal volume. Thinly coated soot aggregates composed of soot core (*a*) and sulfate shell (*R*_*shell*_). It is assumed that soot volumes fractions were 2/3 in the simulations of thinly coated soot aerosols. For the heavily coated cases, the soot aggregates are internally mixed with the sulfate spherical particles (*R*_*sul*_), and their soot volumes fractions were 1/3. The volume-equivalent single core-shell sphere approximations were applied for these aged soot aerosols. It is assumed that the core is soot and the shell is the sulfate. The spherical approximations were calculated using the widely used Lorenz-Mie-Debye theory. The soot refractive index is assumed to be constant (1.95 + 0.79i) for the shortwave spectrum range (0.2–4 μm), and the refractive index of the sulfate particle is assumed to be 1.44^53^,^54^. The morphological and chemical parameters are shown in the Table 1.

### Calculation of black carbon radiative forcing

The superposition T-matrix approach, which uses the numerically exact solution methods to Maxwell’s equations, can be used to calculate the T-matrix descriptions of the light scattering from the cluster with an appropriate superposition technique and analytically obtain the random-orientation cross sections and scattering matrices of these clusters^42^. In the open-source Fortran-coded MSTM version 3.0 program, both external and internal mixtures are applicable, and the only limitation is that the spherical surfaces are not overlapped^43^.

The single scattering albedo (SSA) *ω* = *C*_*sca*_/*C*_*ext*_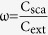 was described for the ratio of the scattering cross section and the extinction cross section. The asymmetry parameter (ASY) was defined as:


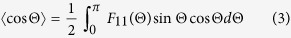


which is the measurement for the entire direction of the light scattering. The value was positive if the forward scattering was dominant, and the value was negative if the backward scattering was dominant. The phase function 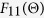

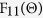
 satisfies the normalization condition with scattering angle 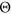:


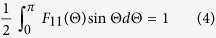


Our approach for investigating the effect of soot aging process on the radiative forcing of solar radiation was to use radiative transfer modeling by the Libradtran package^44^. The model’s DISORT radiative transfer equation solver^55^ was used, and the standard atmospheric profiles for mid-latitude summer was assumed. The air density, pressure, water vapor, and ozone was assumed by the default values. The spectral range was assumed to be 0.2–4 μm, and the band parameterization REPTRAN^56^ was used for the spectral calculations of molecular absorption. The Lambertian surface was assumed constant for all wavelengths. When simulating the irradiances, both the bottom of the atmosphere (BOA, or surface) and top of atmosphere (TOA) was taken into account, and these calculations were performed using a constant day of year of 100 day.

To calculate the radiative forcing of aerosols, the irradiances of clear-sky conditions without aerosols (*F*_*clear*_) and with aerosols (*F*_*aerosol*_) are first simulated. We assumed that the aerosols were distributed in the homogeneous layer of 0–30 km. The altitude of surface (BOA) was considered as 0 km, and the radiative forcing of aerosol (*RF*_*BOA*_) can be obtained by the net downward irradiances with aerosols and without aerosols. The altitude of TOA was assumed to be 120 km, and the radiative forcing of aerosol (*RF*_*TOA*_) was defined as the subtraction of upward irradiance in the clear conditions without aerosols and the aerosol conditions with aerosols.


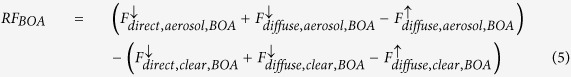



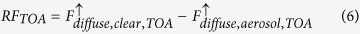


The optical properties of aerosols were calculated for 10 wavelengths in the spectral range of 0.2–4 μm, namely, 0.2, 0.34, 0.44, 0.55, 0.67, 0.87, 1.02, 1.64, 2.1 and 4 μm. These wavelengths are commonly used for the remote sensing of aerosols. In the remote sensing field, the extinction Ångström exponent (EÅE) for a wavelength interval 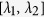 is commonly considered as the qualitative indicator of particle size^16^,^57^.


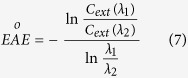


In general, EÅE ≤ 1 indicates size distributions dominated by coarse mode aerosols (radii ≥ 0.5 μm) that are typically associated with dust and sea salt and EÅE ≥ 2 indicates size distributions dominated by fine mode aerosols that are usually associated with urban pollution and biomass burning^58^. In these simulations, according to the aerosols optical depth (*AOD*_0.55_) at *λ*_2_ = 0.55 *μm*, the AOD of different wavelengths (*λ*_1_) can be calculated 
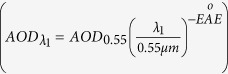
.

## Additional Information

**How to cite this article**: Wu, Y. *et al*. Black carbon radiative forcing at TOA decreased during aging. *Sci. Rep.* **6**, 38592; doi: 10.1038/srep38592 (2016).

**Publisher's note:** Springer Nature remains neutral with regard to jurisdictional claims in published maps and institutional affiliations.

## Figures and Tables

**Table 1:** Morphological parameters of black carbon particles with different aging states.

	Freshly emitted soot	Thinly coated soot	Heavily coated soot
*a* (μm)	0.02	0.02	0.02
*N*_*s*_	200	200	200
*Df*	1.8	2.4	3.0
*F*_*soot*_	1	2/3	1/3
*R*_*sul*_ (μm)	0	0	0.1687
*R*_*shell*_ (μm)	0	0.0229	0

## References

[b1] Cappa C. D. . Radiative absorption enhancements due to the mixing state of atmospheric black carbon. Science 337, 1078–1081 (2012). 22936774 10.1126/science.1223447

[b2] Jacobson M. Z. A physically-based treatment of elemental carbon optics: Implications for global direct forcing of aerosols. Geophysical Research Letters 27, 217–220 (2000).

[b3] Ramanathan V. & Carmichael G. Global and regional climate changes due to black carbon. Nature geoscience 1, 221–227 (2008).

[b4] Bond T. C. . Bounding the role of black carbon in the climate system: A scientific assessment. Journal of Geophysical Research: Atmospheres 118, 5380–5552 (2013).

[b5] Johnson K. S. . Processing of soot in an urban environment: case study from the Mexico City Metropolitan Area. Atmospheric Chemistry and Physics 5, 3033–3043 (2005).

[b6] Ramana M. V. . Warming influenced by the ratio of black carbon to sulphate and the black-carbon source. Nature Geoscience 3, 542–545 (2010).

[b7] Adachi K. & Buseck P. R. Internally mixed soot, sulfates, and organic matter in aerosol particles from Mexico City. Atmospheric Chemistry and Physics 8, 6469–6481 (2008).

[b8] Kahnert M., Nousiainen T., Lindqvist H. & Ebert M. Optical properties of light absorbing carbon aggregates mixed with sulfate: assessment of different model geometries for climate forcing calculations. Optics Express 20, 10042–10058 (2012). 22535095 10.1364/OE.20.010042

[b9] He C. . Variation of the radiative properties during black carbon aging: theoretical and experimental intercomparison. Atmospheric Chemistry and Physics 15, 11967–11980 (2015).

[b10] Lesins G., Chylek P. & Lohmann U. A study of internal and external mixing scenarios and its effect on aerosol optical properties and direct radiative forcing. Journal of Geophysical Research: Atmospheres 107, D10, 4094 (2002).

[b11] Chakrabarty R. K. . Soot superaggregates from flaming wildfires and their direct radiative forcing. Scientific reports 4, 1–8 (2014). 10.1038/srep05508 PMC4076688 24981204

[b12] Bellouin N., Boucher O., Haywood J. & Reddy M. S. Global estimate of aerosol direct radiative forcing from satellite measurements. Nature 438, 1138–1141 (2005). 16372005 10.1038/nature04348

[b13] Chung C. E., Lee K. & Müller D. Effect of internal mixture on black carbon radiative forcing. Tellus B 64, 10925 (2011).

[b14] Haywood J. M. & Ramaswamy V. Global sensitivity studies of the direct radiative forcing due to anthropogenic sulfate and black carbon aerosols. Journal of Geophysical Research: Atmospheres 103, 6043–6058 (1998).

[b15] Park S. H. . Effects of black carbon aging on air quality predictions and direct radiative forcing estimation. Tellus B. 63, 1026–1039 (2011).

[b16] Kahnert M., Nousiainen T. & Lindqvist H. Models for integrated and differential scattering optical properties of encapsulated light absorbing carbon aggregates. Optics express 21, 7974–7993 (2013). 23571889 10.1364/OE.21.007974

[b17] Cheng T., Gu X., Wu Y. & Chen H. Effects of atmospheric water on the optical properties of soot aerosols with different mixing states. Journal of Quantitative Spectroscopy and Radiative Transfer 147, 196–206 (2014).

[b18] Khalizov A. F. . Enhanced light absorption and scattering by carbon soot aerosol internally mixed with sulfuric acid. The Journal of Physical Chemistry A. 113, 1066–1074 (2009). 19146408 10.1021/jp807531n

[b19] Bueno P. A. . Photoacoustic measurements of amplification of the absorption cross section for coated soot aerosols. Aerosol Science and Technology 45, 1217–1230 (2011).

[b20] Kahnert M. & Devasthale A. Black carbon fractal morphology and short-wave radiative impact: a modelling study. Atmospheric Chemistry and Physics 11, 11745–11759 (2011).

[b21] Adachi K., Chung S. H. & Buseck P. R. Shapes of soot aerosol particles and implications for their effects on climate. Journal of Geophysical Research: Atmospheres 115, D15206 (2010).

[b22] Cheng T., Gu X., Wu Y., Chen H. & Yu T. The optical properties of absorbing aerosols with fractal soot aggregates: Implications for aerosol remote sensing. Journal of Quantitative Spectroscopy and Radiative Transfer 125, 93–104 (2013).

[b23] Li J., Pósfai M., Hobbs P. V. & Buseck P. R. Individual aerosol particles from biomass burning in southern Africa: 2, Compositions and aging of inorganic particles. Journal of Geophysical Research: Atmospheres 108, D13 (2003).

[b24] China S., Mazzoleni C., Gorkowski K., Aiken A. & Dubey M. K. Morphology and mixing state of individual freshly emitted wildfire carbonaceous particles. Nature communications 4, 1–7 (2013). 10.1038/ncomms3122 PMC3715871 23824042

[b25] Kahnert M. On the discrepancy between modeled and measured mass absorption cross sections of light absorbing carbon aerosols. Aerosol Science and Technology 44, 453–460 (2010).

[b26] Mishchenko M. I., Liu L. & Mackowski D. W. T-matrix modeling of linear depolarization by morphologically complex soot and soot-containing aerosols. Journal of Quantitative Spectroscopy and Radiative Transfer 123, 135–144 (2013).

[b27] Wu Y., Cheng T., Zheng L. & Chen H. A study of optical properties of soot aggregates composed of poly-disperse monomers using the superposition T-matrix method. Aerosol Science and Technology 49, 941–949 (2015a).

[b28] Schwarz J. P. . Measurement of the mixing state, mass, and optical size of individual black carbon particles in urban and biomass burning emissions. Geophysical Research Letters 35, L13810 (2008).

[b29] Zhang R. . Variability in morphology, hygroscopicity, and optical properties of soot aerosols during atmospheric processing. Proceedings of the National Academy of Sciences 105, 10291–10296 (2008). 10.1073/pnas.0804860105 PMC2478695 18645179

[b30] Coz E. & Leck C. Morphology and state of mixture of atmospheric soot aggregates during the winter season over Southern Asia-a quantitative approach. Tellus B 63, 107–116 (2011).

[b31] Tritscher T. . Changes of hygroscopicity and morphology during ageing of diesel soot. Environmental Research Letters 6, 034026 (2011).

[b32] Worringen A., Ebert M., Trautmann T., Weinbruch S. & Helas G. Optical properties of internally mixed ammonium sulfate and soot particles-a study of individual aerosol particles and ambient aerosol populations. Applied optics 47, 3835–3845 (2008). 18641753 10.1364/ao.47.003835

[b33] Mishchenko M. I. & Dlugach J. M. Adhesion of mineral and soot aerosols can strongly affect their scattering and absorption properties. Optics letters 37, 704–706 (2012). 22344154 10.1364/OL.37.000704

[b34] Kahnert M., Nousiainen T. & Lindqvist H. Review: Model particles in atmospheric optics. Journal of Quantitative Spectroscopy and Radiative Transfer 146, 41–58 (2014).

[b35] Wu Y., Cheng T., Zheng L., Chen H. & Xu H. Single scattering properties of semi-embedded soot morphologies with intersecting and non-intersecting surfaces of absorbing spheres and non-absorbing host. Journal of Quantitative Spectroscopy and Radiative Transfer 157, 1–13 (2015b).

[b36] Wriedt T. A review of elastic light scattering theories. Particle & particle systems characterization 15, 67–74 (1998).

[b37] Mishchenko M. I., Dlugach J. M. & Liu L. Applicability of the effective-medium approximation to heterogeneous aerosol particles. Journal of Quantitative Spectroscopy and Radiative Transfer 178, 284–294 (2016).

[b38] Liu L., Mishchenko M. I. & Arnott W. P. A study of radiative properties of fractal soot aggregates using the superposition T-matrix method. Journal of Quantitative Spectroscopy and Radiative Transfer 109, 2656–2663 (2008).

[b39] Wu Y. . The single scattering properties of the aerosol particles as aggregated spheres. Journal of Quantitative Spectroscopy and Radiative Transfer 113, 1454–1466 (2012).

[b40] Cheng T., Wu Y., Gu X. & Chen H. Effects of mixing states on the multiple-scattering properties of soot aerosols. Optics express 23, 10808–10821 (2015). 25969118 10.1364/OE.23.010808

[b41] Mackowski D. W. & Mishchenko M. I. Calculation of the T matrix and the scattering matrix for ensembles of spheres. Journal of the Optical Society of America A 13, 2266–78 (1996).

[b42] Mackowski D. W. & Mishchenko M. I. A multiple sphere T-matrix Fortran code for use on parallel computer clusters. Journal of Quantitative Spectroscopy and Radiative Transfer 112, 2182–92 (2011).

[b43] Mackowski D. W. A general superposition solution for electromagnetic scattering by multiple spherical domains of optically active media. Journal of Quantitative Spectroscopy and Radiative Transfer 133, 264–270 (2014).

[b44] Mayer B. & Kylling A. Technical note: The libRadtran software package for radiative transfer calculations-description and examples of use. Atmospheric Chemistry and Physics 5, 1855–1877 (2005).

[b45] Emde C. . The libRadtran software package for radiative transfer calculations (Version 2.0). Geoscientific model development discussions 8, 10237–10303 (2015).

[b46] Khalizov A. F. . Role of OH-initiated oxidation of isoprene in aging of combustion soot. Environmental science & technology 47, 2254–2263 (2013). 23379649 10.1021/es3045339

[b47] Cheng T., Wu Y. & Chen H. Effects of morphology on the radiative properties of internally mixed light absorbing carbon aerosols with different aging status. Optics express 22, 15904–15917 (2014). 24977845 10.1364/OE.22.015904

[b48] China S. . Morphology and mixing state of aged soot particles at a remote marine free troposphere site: Implications for optical properties. Geophysical Research Letters 42, 1243–1250 (2015).

[b49] Wu Y. . The single scattering properties of soot aggregates with concentric core–shell spherical monomers. Journal of Quantitative Spectroscopy and Radiative Transfer 135, 9–19 (2014).

[b50] Huang J. . Taklimakan dust aerosol radiative heating derived from CALIPSO observations using the Fu-Liou radiation model with CERES constraints. Atmospheric Chemistry and Physics 9, 4011–4021 (2009).

[b51] Wang W. . Estimation of radiative effect of a heavy dust storm over northwest China using Fu-Liou model and ground measurements. Journal of Quantitative Spectroscopy and Radiative Transfer 122, 114–126 (2013).

[b52] Shupe M. D. & Intrieri J. M. Cloud radiative forcing of the Arctic surface: The influence of cloud properties, surface albedo, and solar zenith angle. Journal of Climate 17, 616–628 (2004).

[b53] Bond T. C. & Bergstrom R. W. Light absorption by carbonaceous particles: an investigative review, Aerosol Science and Technology 40, 27–67 (2006).

[b54] Hess M., Koepke P. & Schult I. Optical properties of aerosols and clouds: The software package OPAC. Bulletin of the American meteorological society 79, 831–844 (1998).

[b55] Buras R., Dowling T. & Emde C. New secondary-scattering correction in DISORT with increased efficiency for forward scattering. Journal of Quantitative Spectroscopy and Radiative Transfer 112, 2028–2034 (2011).

[b56] Gasteiger J. . Representative wavelengths absorption parameterization applied to satellite channels and spectral bands. Journal of Quantitative Spectroscopy and Radiative Transfer 148, 99–115 (2014).

[b57] Wu Y., Cheng T., Zheng L. & Chen H. Optical properties of the semi-external mixture composed of sulfate particle and different quantities of soot aggregates. Journal of Quantitative Spectroscopy and Radiative Transfer 179, 139–148 (2016).

[b58] Schuster G. L., Dubovik O. & Holben B. N. Angstrom exponent and bimodal aerosol size distributions. Journal of Geophysical Research: Atmospheres (1984–2012) 111, D7 (2006).

